# Effect of Belimumab on Preventing *de novo* Renal Lupus Flares

**DOI:** 10.1016/j.ekir.2023.06.021

**Published:** 2023-07-06

**Authors:** Ioannis Parodis, Julius Lindblom, Nursen Cetrez, Leonardo Palazzo, Henri Ala, Frédéric A. Houssiau, Christopher Sjöwall, Brad H. Rovin

**Affiliations:** 1 Division of Rheumatology, Department of Medicine Solna, Karolinska Institutet and Karolinska University Hospital, Stockholm, Sweden; 2 Department of Rheumatology, Faculty of Medicine and Health, Örebro University, Örebro, Sweden; 3 Pôle de Pathologies Rhumatismales Inflammatoires et Systémiques, Institut de Recherche Expérimentale et Clinique, Université Catholique de Louvain and Service de Rhumatologie, Cliniques Universitaires Saint-Luc, Brussels, Belgium; 4 Department of Biomedical and Clinical Sciences, Division of Inflammation and Infection/Rheumatology, Linköping University, Linköping, Sweden; 5 Department of Internal Medicine, The Ohio State University College of Medicine, Columbus, Ohio, USA

**Keywords:** belimumab, flares, kidney disease, lupus nephritis, predictors, systemic lupus erythematosus

## Abstract

**Introduction:**

Belimumab was recently approved for treating lupus nephritis (LN), yet *de novo* LN cases during belimumab treatment given for nonrenal causes have been reported. Identification of reliable signals of impending flare is imperative.

**Methods:**

We evaluated belimumab efficacy in preventing *de novo* renal flares and factors associated with renal flare occurrence in nephritis-naïve patients with systemic lupus erythematosus (SLE) who are receiving add-on belimumab or placebo in 5 phase 3 clinical trials using Cox regression analysis.

**Results:**

Of 1844 eligible patients, 136 (7.4%) developed a *de novo* renal flare during a 52-week long follow-up. Asian origin (Adjusted Hazard Ratio [HR_adj_]: 1.97; 95% confidence interval [CI]: 1.32–2.94; *P* = 0.001), positive baseline anti-double stranded DNA (anti-dsDNA) levels (HR_adj_: 1.32; 95% CI: 1.07–1.63; *P* = 0.008), and increasing mean prednisone dose during follow-up (HR_adj_: 1.03; 95% CI: 1.02–1.04; *P* < 0.001) were associated with *de novo* renal flares. Low-dose intravenous (IV) belimumab (1 mg/kg monthly) yielded a nearly 3-fold lower hazard of *de novo* renal flare (HR_adj_: 0.38; 95% CI: 0.20–0.73; *P* = 0.004). Subcutaneous (SC) belimumab (200 mg weekly) also yielded a lower hazard (HR_adj._: 0.69; 95% CI: 0.54–0.88; *P* = 0.003). The labeled IV dose (10 mg/kg monthly) conferred no clear protection (HR_adj._: 0.74; 95% CI: 0.50–1.09; *P* = 0.127).

**Conclusion:**

We corroborated the substantial vulnerability of the Asian SLE population to renal affliction. Add-on low-dose IV belimumab (1 mg/kg) and SC belimumab appeared protective against renal flares in nephritis-naïve patients with SLE. The approved IV dose (10 mg/kg) yielded no clear protection.

## Graphical abstract

**Figure:**
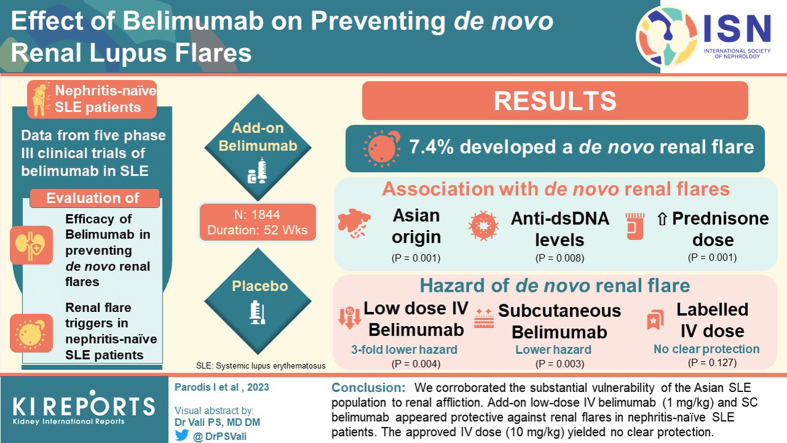

Each LN flare causes a substantial nephron loss, making prompt initiation of therapy and prevention of flares imperative. Identification of readily available signals of impending flare is therefore expected to improve prognosis.

Belimumab is a monoclonal antibody against B cell activating factor belonging to the tumor necrosis factor family that is approved for the treatment of adult^1^, ^2^, ^3^ and pediatric^4^ SLE, and recently approved for active LN.^5^ In light of observed cases of *de novo* LN during belimumab treatment,^6^^,^^7^ we evaluated the efficacy of belimumab in preventing *de novo* renal flares as well as predictors of *de novo* renal flare occurrence in patients with SLE and no prior history of renal disease who are undergoing standard therapy (ST) with or without add-on belimumab in a *post hoc* analysis of data from 5 phase 3 clinical trials.

## Methods

### Study Population

Data from 5 phase 3 clinical trials of belimumab in SLE, including BLISS-52 (intravenous [IV] belimumab, NCT00424476, *N* = 865)^2^, BLISS-76 (IV belimumab, NCT00410384, *N* = 819)^3^, BLISS-NEA (IV belimumab in Northeast Asia, NCT01345253, *N* = 677)^8^, BLISS-SC (SC belimumab, NCT01484496, *N* = 836)^9^, and EMBRACE (belimumab in patients with SLE who are of African ancestry, NCT0163224, *N* = 448)^10^ were utilized. All patients judged eligible to be included in the trials fulfilled the American College of Rheumatology revised criteria for SLE,^11^ were adults, had an antinuclear antibody titer ≥1:80 and/or serum anti-dsDNA antibody level ≥30 IU/ml at screening, and a Safety of Estrogens in Lupus National Assessment-Systemic Lupus Erythematosus Disease Activity Index^12^ score ≥6 (BLISS-52 and BLISS-76) or ≥8 (BLISS-SC, BLISS-NEA, or EMBRACE). All patients were on stable nonbiological ST for ≥30 days before the baseline of the double-blinded phase. Progressive restrictions were imposed during the trial periods on concurrent medications as well as on glucocorticoid intake. Patients with severe active central nervous system involvement or severe active LN were excluded. Patients were randomized to receive belimumab 1 mg/kg, belimumab 10 mg/kg, or placebo in addition to ST for 52 weeks in BLISS-52 and for 76 weeks in BLISS-76. Belimumab and placebo were administered IV at baseline, week 2, week 4, and thereafter every fourth week until week 48 in BLISS-52 and until week 72 in BLISS-76. In BLISS-NEA and EMBRACE, patients received either belimumab 10 mg/kg or placebo in addition to ST. In BLISS-SC, patients received weekly doses of belimumab 200 mg or placebo, administered SC in addition to ST.

The study population comprised 1844 patients with no history of or current renal involvement as determined by a baseline renal British Isles Lupus Assessment Group (BILAG)^13^ score E, a baseline renal SLE Disease Activity Index 2000 (SLEDAI-2K)^14^ score of zero (with renal SLEDAI-2K defined as the sum of the renal descriptors of the index i.e., proteinuria, hematuria, pyuria, and urinary casts), and a urinary protein-to-creatinine ratio <0.5 mg/mg.

The Preferred Reporting Items for Systematic review and Meta-Analyses of Individual Participant Data checklist and flow diagram are provided in the online Supplementary Material.

### Data Management

To extract variables, we followed the instructions in the dictionary provided to find the same variable of interest in the original datasets of the 5 different studies. For each variable, homogenization to the same unit across studies was performed. In the case of missing values, the last observation carried forward methodology was applied for data imputation.

### Clinical Definitions

BILAG is a composite index intended for the assessment of disease activity in SLE, which was developed according to the physician’s intention to treat.^13^ The instrument assigns separate alphabetic scores to each one of 8 organ system domains: a BILAG score A denotes active disease requiring intensification of the therapy, a BILAG score B denotes less active disease than score A requiring only symptomatic therapy, a BILAG score C indicates stable and mild disease, a BILAG score D indicates no current but history of involvement in the respective organ system, and a BILAG score E indicates no history of or current involvement in the respective organ system. Using the proposed definition of SLE flares measured by the BILAG index,^15^ *de novo* renal flares were defined as a change from renal BILAG E to a renal BILAG A or B within 52 weeks of follow-up. A renal BILAG A score (first flare occurrence) requires 2 or more of the following criteria to have been present during the previous month, unless noted otherwise, provided that 1, 4, or 5 is included: (i) proteinuria (24-hour urinary protein >1 g or urinary dipstick 3 or more); (ii) accelerated hypertension; (iii) creatinine clearance <50 ml/min; (iv) active urinary sediment, pyuria (>5 white blood cells/high power field), hematuria (>5 red blood cells/high power field), or red cell casts, in the absence of infection; and (v) histological evidence of active nephritis within the last 3 months. A renal BILAG B score (first flare occurrence) requires one of the following criteria to have been present during the previous month: (i) 1 of the renal BILAG A criteria; (ii) urinary dipstick 2+ or more; or (iii) 24-hour urinary protein >0.5 g but <1 g.

SLEDAI is an index used for the measurement of global SLE disease activity; this instrument was developed to account for the physician’s global judgment and contains 24 descriptors, each scored separately according to a descriptor-specific weight.^16^ SLEDAI-2K was later developed as a modified version of the SLEDAI that counts the descriptors proteinuria, rash, alopecia, and mucous membrane lesions as active at any time they are present, in contrast to the SLEDAI in which they are counted as active only at their first occurrence or upon recurrence.^14^ In the present study, global SLE disease activity was assessed using the SLEDAI-2K, and clinical disease was assessed using the clinical version of SLEDAI-2K (cSLEDAI-2K)^17^ that is, the SLEDAI-2K score after exclusion of the serological descriptors (DNA binding and hypocomplementemia). Organ damage was assessed using the Systemic Lupus International Collaborating Clinics / American College of Rheumatology Damage Index.^18^

### Serological Markers

Serological markers investigated as potential predictors of *de novo* renal flare included anti-dsDNA positivity (≥30 IU/ml); anti-Smith (Sm) positivity (≥15.0 IU/ml); anti-ribonucleoprotein positivity (≥25 IU/ml); anti-ribosomal protein P positivity (≥12.0 IU/ml in BLISS-52; ≥12.4 IU/ml in BLISS-76; ≥12.5 IU/ml in BLISS-SC); antiphospholipid positivity; anticardiolipin immunoglobulin (Ig)A (≥10.0 IU/ml in BLISS-52 and BLISS-76; ≥11.0 IU/ml in BLISS-NEA, BLISS-SC, and EMBRACE); anticardiolipin IgG (≥10.0 IU/ml in BLISS-52 and BLISS-76; ≥14.0 IU/ml in BLISS-NEA, BLISS-SC, and EMBRACE) and anticardiolipin IgM (≥10.0 IU/ml in BLISS-52 and BLISS-76; ≥12.0 IU/ml in BLISS-NEA, BLISS-SC, and EMBRACE); anti-***β***2-glycoprotein I (***β***2-GPI) IgA, IgG, and IgM (≥21.0 IU/ml for all Ig isotypes); lupus anticoagulant positivity (≥45.0 IU/ml in BLISS-SC, ≥41.0 IU/ml in EMBRACE); low levels of C3 (<90.0 mg/dl); low levels of C4 (<16.0 mg/dl in BLISS-52 and BLISS-76; <10.0 mg/dl in BLISS-NEA, BLISS-SC, and EMBRACE); and levels of B cell activating factor belonging to the tumor necrosis factor family.

### Statistical Analysis

Descriptive statistics are reported as numbers (percentage) or means (standard deviation), and medians (interquartile range) are indicated in case of nonnormal distributions. For comparisons between patients who developed *de novo* renal flare and patients who did not, the nonparametrical Mann-Whitney *U* test or Kruskal-Wallis test was used for continuous variables, and the Pearson’s chi-square (*χ*^2^) test was used for binomial variables, as appropriate. Predictors of renal flare occurrence were investigated using univariable and multivariable shared frailty Cox models accounting for trial effects. Reverse Kaplan-Meier survival curves were used to illustrate the time to first *de novo* renal flare, and the log-rank test was used for unadjusted comparisons between belimumab and placebo recipients. *P* values <0.05 were deemed statistically significant.

Variables in the multivariable Cox regression analysis included variables deemed clinically important (age, sex, ethnicity, serum creatinine, and urinary protein-to-creatinine ratio) and variables with sufficient available values (<5% missing data) that reached statistical significance (*P* < 0.05) in univariable analysis. Results from Cox regression analysis are presented as the coefficient, HR, 95% CI and *P*-value.

All analyses were performed, and all illustrations were developed using the R Statistical Software version 4.2.1 (R Foundation for Statistical Computing, Vienna, Austria).

## Results

A total of 1844 patients formed the population of the present study. Patient characteristics are shown in Table 1 and are stratified by treatment arms in Supplementary Tables S1 to S4. A total of 136 patients (7.4%) developed at least 1 *de novo* renal flare during follow-up (13 BILAG A; 123 BILAG B). Among those, greater proportions were of Asian origin (31.6% in the flare group vs. 20.7% in the no-flare group; *P* = 0.004) and had positive anti-dsDNA levels at baseline (74.3% vs. 61.6%; *P* = 0.004) and/or low baseline levels of C3 (51.5% vs. 38.8%; *P* = 0.005) or C4 (47.1% vs. 36.4%; *P* = 0.017) compared with patients who did not develop renal SLE.

**Table 1:** Patient characteristics and comparisons between patients who developed at least 1 renal flare and patients who did not from baseline through week 52

Patient characteristics	All patients (*N* = 1844)	Renal flare (*n* = 136)	No renal flare (*n* = 1708)	*P* value
Demographics				
Age; mean (SD)	38.6 (11.8)	36.7 (11.7)	38.7 (11.8)	0.035^a^
Female sex; *n* (%)	1769 (96.0)	132 (97.1)	1637 (95.8)	0.642
Ancestries; *n* (%)				
Asian	396 (21.5)	43 (31.6)	353 (20.7)	0.004^a^
Black/African American	339 (18.4)	24 (17.6)	315 (18.4)	0.908
Indigenous American^b^	243 (13.2)	19 (14.0)	224 (13.1)	0.879
White/Caucasian	866 (47.0)	50 (36.8)	816 (47.8)	0.017^a^
Clinical data at baseline				
SLE duration (yrs); median (IQR)	4.1 (1.4–9.1); *N* = 1843	3.3 (1.2–8.1)	4.2 (1.4–9.1); *n* = 1707	0.260
Mean BMI (week 0–52); mean (SD)	26.3 (6.4); *N* = 1793	25.2 (5.8); *n* = 126	26.3 (6.4); *n* = 1667	0.052
SLEDAI-2K; mean (SD)	9.6 (3.0)	9.1 (3.2)	9.5 (2.9)	0.031^a^
Extra renal cSLEDAI-2K; mean (SD)	7.2 (2.9)	6.5 (3.0)	7.3 (2.9)	< 0.001^a^
SDI score; median (IQR)	0.0 (0.0–1.0); *N* = 1842	0.0 (0.0–1.0)	0.0 (0.0–1.0); *n* = 1706	0.829
SDI score ≥1; *n* (%)	675 (36.6); *N* = 1842	49 (36.0)	626 (36.7); *n* = 1706	0.950
Serological profile at baseline				
Anti-dsDNA (+); *n* (%)	1153 (62.5)	101 (74.3)	1052 (61.6)	0.004^a^
Anti-Sm (+); *n* (%)				
at baseline	348 (24.3); *N* = 1433	31 (33.3); *n* = 93	317 (23.7); *n* = 1340	0.048^a^
ever	251 (24.5); *N* = 1435	31 (33.3); *n* = 93	320 (23.8); *n* = 1342	0.053
Anti-RNP (+); *n* (%)				
at baseline	206 (29.1); *N* = 709	20 (42.6); *n* = 47	186 (28.1); *n* = 662	0.052
ever	207 (29.1); *N* = 712	20 (41.7); *n* = 48	187 (28.2); *n* = 664	0.068
Anti-ribosomal P (+); *n* (%)				
at baseline	463 (28.6); *N* = 1617	41 (37.3); *n* = 110	422 (28.0); *n* = 1507	0.049^a^
ever	463 (28.6); *N* = 1617	41 (37.3); *n* = 110	422 (28.0); *n* = 1507	0.049^a^
aPL (+); *n* (%)				
aCL				
aCL IgA	34 (1.9); *N* = 1781	6 (4.5); *n* = 134	28 (1.7); *n* = 1647	0.053
aCL IgG	243 (13.6); *N* = 1784	28 (20.9); *n* = 134	215 (13.0); *n* = 1650	0.015^a^
aCL IgM	182 (10.2); *N* = 1784	14 (10.4); *n* = 134	168 (10.2); *n* = 1650	1.000
Anti-***β***_2_-GPI				
anti-***β***_2_-GPI IgA	117 (16.4); *N* = 714	12 (25.0); *n* = 48	105 (15.8); *n* = 666	0.142
anti-***β***_2_-GPI IgG	23 (3.2); *N*=714	2 (4.2); *n* = 48	21 (3.2); *n* = 666	1.000
anti-***β***_2_-GPI IgM	49 (6.9); *N* = 714	3 (6.2); *n* = 48	46 (6.9); *n* = 666	1.000
LAC	134 (19.0); *N* = 706	5 (10.4); *n* = 48	129 (19.6); *n* = 658	0.169
aPL (+) ever	674 (37.7); *N* = 1788	58 (43.3); *n* = 134	616 (37.2); *n* = 1654	0.195
BAFF (ng/ml); mean (SD)	1.48 (1.04); *N* = 1617	1.40 (0.89); *n* = 116	1.49 (1.05); *n* = 1501	0.274
Low C3 levels; *n* (%)	732 (39.7)	70 (51.5)	662 (38.8)	0.005^a^
Low C4 levels; *n* (%)	686 (37.2)	64 (47.1)	622 (36.4)	0.017^a^
Renal markers at baseline				
Serum albumin (g/l); mean (SD)	41.51 (3.70)	40.91 (4.00)	41.56 (3.67)	0.045^a^
Serum creatinine (***μ***mol/l); mean (SD)	66.55 (14.21)	67.14 (18.09)	66.51 (13.86)	0.259
eGFR (ml/min); mean (SD)	113.32 (35.3); *N* = 1843	111.81 (37.3)	113.44 (35.1); *n* = 1707	0.732
UPCR (mg/mg); mean (SD)	0.12 (0.07)	0.13 (0.08)	0.12 (0.07)	0.134
Medications				
Prednisone equivalent dose during follow-up (mg/d); mean (SD)	9.95 (7.86)	12.67 (9.26)	9.73 (7.70)	< 0.001^a^
Treatment at baseline; n (%)	*N* = 1633	*n* = 118	*n* = 1515	
Antimalarial agents^C^	1258 (68.2)	89 (65.4)	1169 (68.4)	0.530
Immunosuppressants				
Azathioprine	352 (19.1)	22 (16.2)	330 (19.3)	0.433
Methotrexate	278 (15.1)	11 (8.1)	267 (15.6)	0.025^a^
Mycophenolic acid	151 (8.2)	24 (17.6)	127 (7.4)	< 0.001^a^
Oral cyclophosphamide	21 (1.1)	6 (4.4)	15 (0.9)	0.001^a^
Tacrolimus	40 (2.2)	2 (1.5)	38 (2.2)	0.783
Cyclosporine	53 (2.9)	4 (2.9)	49 (2.9)	1.000
Leflunomide	43 (2.3)	6 (4.4)	37 (2.2)	0.169
Trial intervention; *n* (%)				
Placebo	588 (31.9)	57 (41.9)	531 (31.1)	0.012^a^
Belimumab	1256 (68.1)	79 (58.1)	1177 (68.9)	0.012^a^
IV 1 mg/kg	311 (16.9)	13 (9.6)	298 (17.4)	0.025^a^
IV 10 mg/kg	602 (32.6)	48 (35.3)	554 (32.4)	0.556
SC 200 mg	343 (18.6)	18 (13.2)	325 (19.0)	0.120
Belimumab approved dose; *n* (%)	945 (61.6); *N* = 1533	66 (53.7); *n* = 123	879 (62.3); *n* = 1410	0.071

(+), positive levels; aCL, anticardiolipin antibodies; anti-***β***_2-_GPI, anti-***β***2-glycoprotein I antibodies; anti-dsDNA, anti-double-stranded DNA antibodies; anti-RNP, anti-ribonucleoprotein antibodies; anti-Sm, anti-Smith antibodies; aPL, antiphospholipid antibodies; BAFF, B cell activating factor belonging to the TNF ligand family; BMI, body mass index; C3, complement component 3; C4, complement component 4; cSLEDAI-2K, clinical SLEDAI-2K; eGFR, estimated glomerular filtration rate; Ig, immunoglobulin; IQR, interquartile range; IV, intravenous; LAC, lupus anticoagulant; SC, subcutaneous; SDI, Systemic Lupus International Collaborating Clinics (SLICC)/American College of Rheumatology (ACR) Damage Index; SLE, systemic lupus erythematosus; SLEDAI-2K, Systemic Lupus Erythematosus Disease Activity Index 2000; UPCR, urinary protein-to-creatinine ratio.

Data are presented as numbers (%) or means (SD). In case of nonnormal distributions, the medians (interquartile range) are indicated. In case of missing values, numbers of patients with available data are indicated.

a Statistically significant *P* values.

b Alaska Native or American Indian from North, South or Central America.

c Hydroxychloroquine, chloroquine, mepacrine, mepacrine hydrochloride or quinine sulfate.

Of 1844 study participants, 602 received IV belimumab 10 mg/kg (BLISS-52: *n* = 151, BLISS-76: *n* = 170, BLISS-NEA: *n* = 128, and EMBRACE: *n* = 153), 311 received IV belimumab 1 mg/kg (BLISS-52: *n* = 137, BLISS-76: *n* = 174), 343 received SC belimumab (BLISS-SC: *n* = 343), and 588 study participants received placebo (BLISS-52: *n* = 131, BLISS-76: *n* = 169, BLISS-NEA: *n* = 61, BLISS-SC: *n* = 169, and EMBRACE: *n* = 58). Patient characteristics across belimumab dosage forms are shown in Supplementary Table S5. For comparisons between belimumab and placebo, placebo-treated patients in the respective trials formed the comparator groups to account for the original randomization.

**Figure 1:**
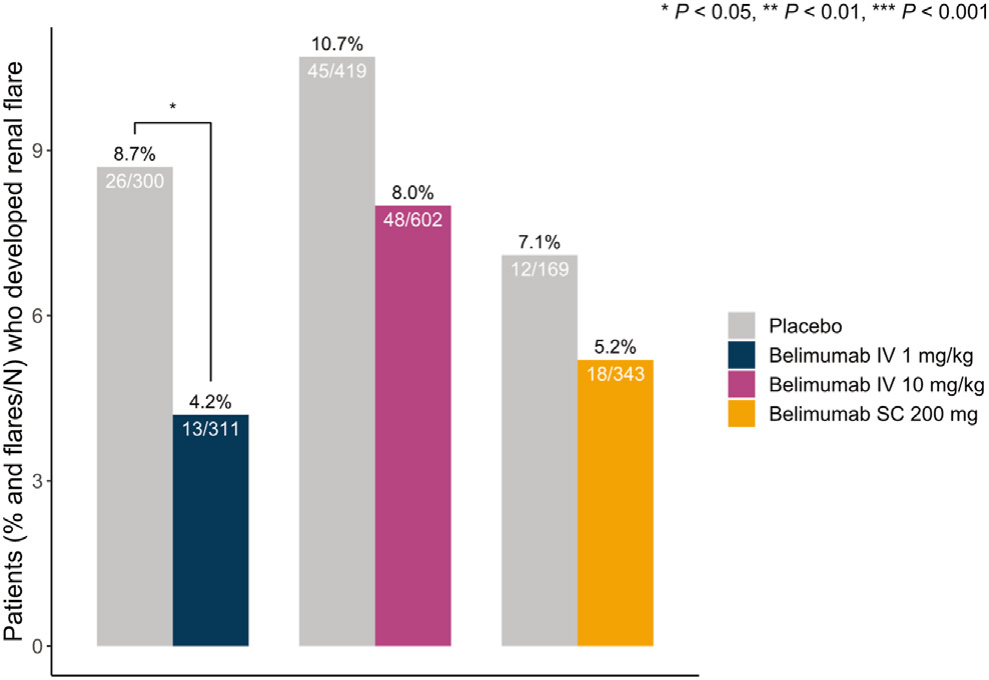
Renal flares in patient subgroups across belimumab dosage forms. Bars depicting proportions of patients who developed at least 1 *de novo* renal flare during follow-up in patient subgroups exposed to belimumab treatment of different dosage forms compared with patients from the same studies treated with placebo. IV, intravenous; SC, subcutaneous.

**Figure 2:**
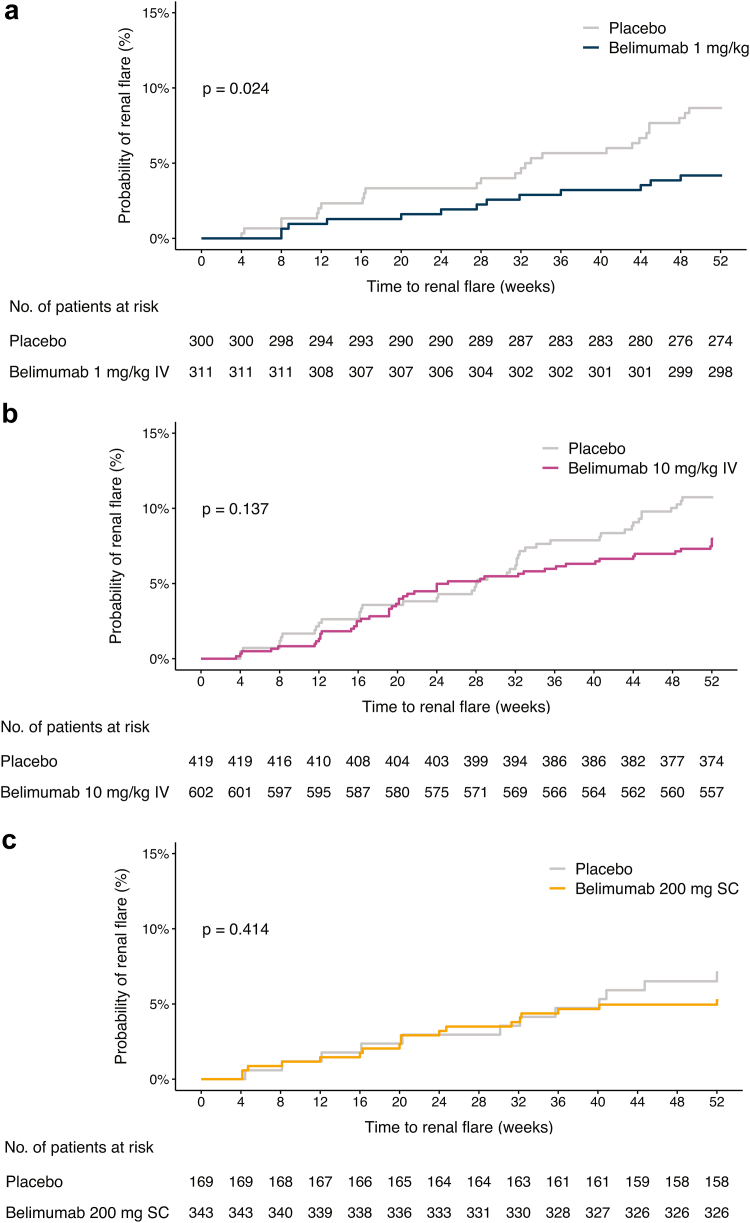
Belimumab dosage forms in relation to development of at least 1 renal flare. The graphs in the upper panels (reverse Kaplan-Meier survival curves) delineate proportions of patients who developed a *de novo* renal flare over the course of the study period, stratified into patients who received (a) IV belimumab 1 mg/kg, (b) IV belimumab 10 mg/kg, or (c) SC belimumab 200 mg, and placebo-recipients. The lower panels (risk tables) show numbers of participants in the 2 groups over time, decreasing due to documentation of at least 1 renal flare. IV, intravenous; SC, subcutaneous.

A lower proportion of patients who received IV belimumab 1 mg/kg (nonlicensed dose) developed *de novo* renal flares (13/311; 4.2%) compared with placebo-treated patients in the same studies (26/300; 8.7%; *P* = 0.036). The proportion of patients who received IV belimumab 10 mg/kg (licensed dose; 48/602, 8.0% vs. 45/419, 10.7%; *P* = 0.161) or SC belimumab 200 mg (licensed dose; 18/343, 5.2% vs. 12/169, 7.1%; *P* = 0.523) and developed *de novo* renal flares did not differ from the proportion of placebo-treated patients in the same studies who developed a *de novo* renal flare (Figure 1). Reverse Kaplan-Meier survival curves illustrating the time to first *de novo* renal flare among belimumab versus placebo recipients are shown in Figure 2.

**Figure 3:**
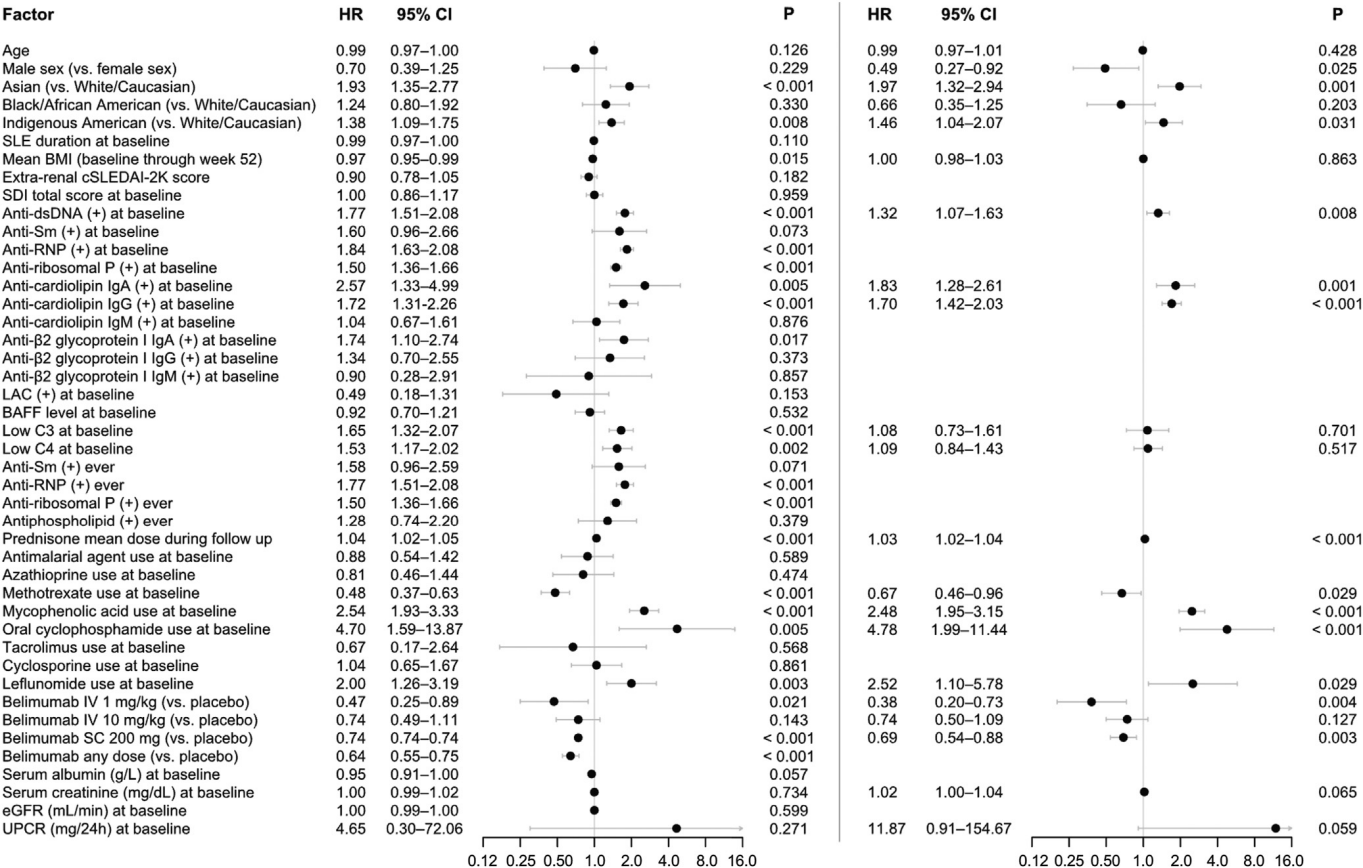
Factors associated with *de novo* renal flare development. Forest plots illustrating results from univariable (left) and multivariable (right) Cox regression analysis, investigating predictors of *de novo* renal flare development. (+), positive levels; anti-dsDNA, anti-double-stranded DNA antibodies; anti-RNP, anti-ribonucleoprotein antibodies; anti-Sm, anti-Smith antibodies; BAFF, B cell activating factor belonging to the TNF ligand family; BMI, body mass index; C3, complement component 3; C4, complement component 4; cSLEDAI-2K, clinical Systemic Lupus Erythematosus Disease Activity Index 2000; CI, confidence interval; eGFR, estimated glomerular filtration rate; HR, hazard ratio; Ig, immunoglobulin; IV, intravenous; LAC, lupus anticoagulant; SC, subcutaneous; SDI: Systemic Lupus International Collaborating Clinics (SLICC)/American College of Rheumatology (ACR) Damage Index; SLE, systemic lupus erythematosus; UPCR, urinary protein-to-creatinine ratio.

To account for the time to the first renal flare occurrence, we used a shared frailty Cox model adjusting for trial effects. Results are illustrated in Figure 3. In univariable analysis, belimumab use (any dose) yielded a lower hazard of renal flare occurrence compared to placebo (HR: 0.64; 95% CI: 0.55–0.75; *P* < 0.001). This held true for the subgroup receiving IV belimumab 1 mg/kg (HR: 0.47; 95% CI: 0.25–0.89; *P* = 0.021) and the subgroup receiving SC belimumab 200 mg (HR: 0.74; 95% CI: 0.74–0.74; *P* < 0.001). However, albeit pointing toward a benefit, the association between IV belimumab 10 mg/kg and *de novo* renal flare occurrence did not reach statistical significance (HR: 0.74; 95% CI: 0.49–1.11; *P* = 0.143).

In multivariable Cox regression analysis adjusting for age, sex, ethnicity, serum creatinine, urinary protein-to-creatinine ratio, and variables that reached statistical significance (*P* < 0.05) in univariable analysis, Asian origin (HR: 1.97; 95% CI: 1.32–2.94; *P* = 0.001), positive levels of anti-dsDNA (HR: 1.32; 95% CI: 1.07–1.63; *P* = 0.008), anticardiolipin IgA (HR: 1.83; 95% CI: 1.28–2.61; *P* = 0.001) and anticardiolipin IgG (HR: 1.70; 95% CI: 1.42–2.03; *P* < 0.001) at baseline, and increasing mean prednisone dose from baseline until renal flare occurrence versus throughout the follow-up if no renal flare occurred (HR: 1.03; 95% CI: 1.02–1.05; *P* < 0.001) were associated with *de novo* renal flare occurrence, whereas male sex showed a negative association with *de novo* renal flares (HR: 0.49; 95% CI: 0.27–0.92; *P* = 0.025), among other factors (Figure 3).

Importantly, low-dose IV belimumab (1 mg/kg) yielded a nearly 3-fold lower hazard of *de novo* renal flare occurrence (HR: 0.38; 95% CI: 0.20–0.73; *P* = 0.004) and SC belimumab (200 mg weekly) also yielded a lower hazard (HR: 0.69; 95% CI: 0.54–0.88; *P* = 0.003). However, despite a numerically lower hazard, the labeled dose of IV belimumab (10 mg/kg) conferred no statistically significant protection (HR: 0.74; 95% CI: 0.50–1.09; *P* = 0.127; Figure 3).

## Discussion

Prompted by reports of *de novo* LN cases during treatment with belimumab,^6^^,^^7^ we studied frequencies and predictors of *de novo* renal flare in patients with SLE and no nephritis history treated with belimumab or placebo in addition to nonbiological ST (antimalarials, immunosuppressants, and glucocorticoids) for active extrarenal disease in clinical trial settings. Interestingly, add-on IV belimumab 1 mg/kg yielded the greatest protection against renal flare development across belimumab dosage forms, whereas no clear protection was documented for the approved IV dose.

Recently, add-on IV belimumab 10 mg/kg yielded superiority to placebo in addition to standard induction therapy regimens along with an acceptable safety profile in the phase 3 BLISS-LN clinical trial of patients with SLE who were treated for active LN.^5^ Moreover, in observational studies^19^^,^^20^ as well as in an early *post hoc* analysis of the BLISS-52 and BLISS-76 trials of belimumab in active extrarenal SLE,^21^ belimumab use showed promise in terms of treating renal SLE and protecting against renal flares. However, the occurrence of *de novo* LN during treatment with the approved dose of belimumab^6^^,^^7^ suggests that belimumab may not be suitable for all patients and evinces a need for personalized treatment approaches. The latter becomes urgent in the era of trial successes for SLE and LN, and an enriched armamentarium of therapeutic choices, but indistinct indicators of which treatment would best suit which patient.

In our previous report of *de novo* LN cases during belimumab therapy, we speculated that activation of LN may be due to inhibition of regulatory B cell subsets by belimumab.^6^ Although no experimental proof of this speculation exists to date, this idea is supported by our previous observation that serum interleukin-10 decreases rapidly, prominently, and in a sustained fashion upon initiation of belimumab treatment.^22^ *De novo* LN flare contrasts with the recent approval of belimumab as an add-on therapy for active LN. However, it is important to keep in mind that patients with active LN suffer significant urinary protein losses, including a portion of administered drug.^23^ Together with the present study showing that low-dose belimumab protects against *de novo* LN flares, we suggest that lower belimumab doses than those currently licensed warrant investigation for the treatment of patients with SLE and low-grade or no proteinuria. In this respect, results from a currently ongoing investigator-initiated trial of IV belimumab 120 mg in China^24^ are awaited.

We also demonstrated that patients with SLE who are of Asian origin were more prone to develop *de novo* renal flares compared to Caucasian or White patients. Moreover, Cox regression analysis accounting for the time to the first documented flare revealed the importance of positive anti-dsDNA and anticardiolipin (IgA and IgG) as indicators of impending renal flares.

Limitations of the present study include its *post hoc* nature and selection bias imposed by the trial criteria, impeding the applicability of findings to real world populations. Moreover, renal flares were not ascertained with a kidney biopsy. Nevertheless, this is to the authors’ knowledge the largest study investigating the protection conferred from belimumab against *de novo* renal flares in a nonrenal SLE population.

## Conclusion

In summary, patients of Asian origin appeared particularly susceptible to new onset renal involvement, corroborating the substantial vulnerability of the Asian SLE population to renal affliction. Add-on low-dose IV 1 mg/kg and SC 200 mg belimumab in addition to nonbiological ST appeared protective against renal flares in patients with SLE who have no prior history of nephritis, whereas addition of the approved IV dose (10 mg/kg) yielded no clear protection. Lack of protection is not to be considered equal to predisposition and in this respect, it is important to underscore that the approved IV belimumab dose did not appear to predispose to renal flare, rendering reassurance. The discrepant results between low and approved IV belimumab doses warrant in-depth mechanistic exploration of underlying reasons such as potential inhibitory effects of belimumab on B cell subsets with regulatory properties.

## Disclosure

IP has received research funding and/or honoraria from Amgen, AstraZeneca, Aurinia Pharmaceuticals, Elli Lilly, Gilead, GlaxoSmithKline, Janssen, Novartis, Otsuka, and Roche. BHR is a consultant and medical advisor for GlaxoSmithKline. The other authors declare no conflicts of interest.

## Supplementary Material

Supplementary File (PDF)

**Table S1.** Comparisons between patients who developed at least 1 renal flare and patients who did not during follow-up in the subgroup of patients treated with IV belimumab 1 mg/kg.

**Table S2.** Comparisons between patients who developed at least 1 renal flare and patients who did not during follow-up in the subgroup of patients treated with IV belimumab 10 mg/kg.

**Table S3.** Comparisons between patients who developed at least 1 renal flare and patients who did not during follow-up in the subgroup of patients treated with SC belimumab 200 mg.

**Table S4.** Comparisons between patients who developed at least 1 renal flare and patients who did not during follow-up in the subgroup of placebo recipients.

**Table S5.** Demographics and comparisons in the subgroup of belimumab-treated patients, stratified by belimumab dosage forms.

**Preferred Reporting Items for Systematic review and Meta-Analyses (PRISMA) Checklist.**

**Preferred Reporting Items for Systematic review and Meta-Analyses (PRISMA) Flow Diagram.**
