# Supplementary material for: Effect of Belimumab on Preventing de novo Renal Lupus Flares
Source: Kidney Int Rep. 2023 Jul 6;8(9):1822–30. doi: 10.1016/j.ekir.2023.06.021 (PMC10496079; doi:10.1016/j.ekir.2023.06.021)
Supplement: Supplementary File (PDF) [file mmc1.pdf]

Supplementary Material

Table of Contents

SUPPLEMENTARY TABLES 2

Supplementary Table S1. Comparisons between patients who developed at least one renal flare and patients who did not during follow-up in the subgroup of patients treated with IV belimumab 1 mg/kg. 2

Supplementary Table S2. Comparisons between patients who developed at least one renal flare and patients who did not during follow-up in the subgroup of patients treated with IV belimumab 10 mg/kg. 4

Supplementary Table S3. Comparisons between patients who developed at least one renal flare and patients who did not during follow-up in the subgroup of patients treated with SC belimumab 200 mg. 6

Supplementary Table S4. Comparisons between patients who developed at least one renal flare and patients who did not during follow-up in the subgroup of placebo recipients. 8

Supplementary Table S5. Demographics and comparisons in the subgroup of belimumab-treated patients, stratified by belimumab dosage forms. 10

SUPPLEMENTARY TABLES

Supplementary Table S1. Comparisons between patients who developed at least one renal flare and patients who did not during follow-up in the subgroup of patients treated with IV belimumab 1 mg/kg.

	All patients (N=311)	Renal flare (N=13)	No renal flare (N=298)	*P* value
**Patient characteristics**				
Age; mean (s.d.)	39.2 (11.6)	36.9 (9.3)	39.3 (11.7)	0.500
Female sex; n (%)	294 (94.5)	13 (100.0)	281 (94.3)	0.793
Ancestries; n (%)				
Asian	41 (13.2)	3 (23.1)	38 (12.8)	0.510
Black/African American	27 (8.7)	0 (0.0)	27 (9.1)	0.527
Indigenous American*	65 (20.9)	5 (38.5)	60 (20.1)	0.214
White/Caucasian	178 (57.2)	5 (38.5)	173 (58.1)	0.266
SLE duration (years); median (IQR)	4.5 (1.1–9.2)	1.6 (0.6–3.7)	4.6 (1.2–9.5)	0.068
Mean BMI (week 0–52); mean (s.d.)	26.6 (6.4)	25.9 (7.7)	26.6 (6.4)	0.450
SLEDAI-2K; mean (s.d.)	9.2 (3.3)	8.5 (1.9)	9.3 (3.3)	0.470
Extra renal cSLEDAI-2K; mean (s.d.)	6.8 (3.0)	6.2 (1.5)	6.8 (3.0)	0.469
SDI score; median (IQR)	0.0 (0.0–1.0); N=310	0.0 (0.0–1.0)	0.0 (0.0–1.0); N=297	0.929
SDI score ≥1; n (%)	130 (41.9); N=310	6 (46.2)	124 (41.8); N=297	0.978
Anti-dsDNA (+); n (%)	191 (61.4)	8 (61.5)	183 (61.4)	1.000
Anti-Sm (+); n (%)				
at baseline	76 (24.4)	3 (23.1)	73 (24.5)	1.000
ever	76 (24.4)	3 (23.1)	73 (24.5)	1.000
Anti-RNP (+); n (%)				
at baseline	NA	NA	NA	NA
ever	NA	NA	NA	NA
Anti-ribosomal P (+); n (%)				
at baseline	108 (35.4); N=305	6 (50.0); N=12	102 (34.8); N=293	0.441
ever	108 (35.4); N=305	6 (50.0); N=12	102 (34.8): N=293	0.441
aPL (+); n (%)				
aCL				
aCL IgA	3 (1.0); N=309	0 (0.0)	3 (1.0); N=296	**0.014**
aCL IgG	69 (22.2)	7 (53.8)	60 (20.8)	0.598
aCL IgM	24 (7.7)	2 (15.4)	22 (7.4)	**0.001**
Anti-β_2-GPI				
anti-β_2-GPI IgA	NA	NA	NA	NA
anti-β_2-GPI IgG	NA	NA	NA	NA

anti-β_2-GPI IgM	NA	NA	NA	NA
LAC	NA	NA	NA	NA
aPL (+) ever	81 (26.0)	9 (69.2)	72 (24.2)	**0.001**
BAFF (ng/mL); mean (s.d.)	1.64 (1.20); N=309	1.59 (1.22); N=12	1.64 (1.20); N=297	0.603
Low C3 levels; n (%)	125 (40.2)	5 (38.5)	120 (40.3)	1.000
Low C4 levels; n (%)	160 (51.4)	7 (53.8)	153 (51.3)	1.000
Serum albumin (g/L); mean (s.d.)	41.21 (3.83)	40.85 (3.13)	41.23 (3.86)	0.661
Serum creatinine (μmol/L); mean (s.d.)	70.17 (13.37)	61.54 (9.08)	70.54 (13.41)	**0.009**
UPCR (mg/mg); mean (s.d.)	0.12 (0.07)	0.11 (0.04)	0.12 (0.07)	0.561
eGFR (mL/min); mean (s.d.)	107.25 (32.64)	107.15 (20.76)	107.26 (33.09)	0.633
Prednisone equivalent dose during follow-up	9.59 (7.70)	10.79 (8.79)	9.54 (7.67)	0.628
Treatment at baseline; n (%)				
Antimalarial agents[†]	200 (64.3)	7 (53.8)	193 (64.8)	0.611
Immunosuppressants				
Azathioprine	64 (20.6)	1 (7.7)	63 (21.1)	0.410
Methotrexate	53 (17.0)	1 (7.7)	52 (17.4)	0.590
Mycophenolic acid	21 (6.8)	0 (0.0)	21 (7.0)	0.670
Oral cyclophosphamide	5 (1.6)	0 (0.0)	5 (1.7)	1.000
Tacrolimus	4 (1.3)	0 (0.0)	4 (1.3)	1.000
Cyclosporine	9 (2.9)	1 (7.7)	8 (2.7)	0.834
Leflunomide	7 (2.3)	1 (7.7)	6 (2.0)	0.692

Data are presented as numbers (percentage) or means (standard deviation). In case of non-normal distributions, the medians (interquartile range) are indicated. In case of missing values, numbers of patients with available data are indicated. Statistically significant *P* values are in bold. Data on anti-RNP, anti-β_2-GPI, and LAC positivity were not available for this population.

(+): positive levels; aCL: anti cardiolipin antibodies; anti-β_2-GPI: anti-β2-glycoprotein I antibodies; anti-dsDNA: anti-double-stranded DNA antibodies; anti-RNP: anti-ribonucleoprotein antibodies; anti-Sm: anti-Smith antibodies; aPL: antiphospholipid antibodies; BAFF: B cell activating factor belonging to the TNF ligand family; BMI: body mass index; C3: complement component 3; C4: complement component 4; cSLEDAI-2K: clinical SLEDAI-2K; eGFR: estimated glomerular filtration rate; Ig: immunoglobulin; IQR: interquartile range; IV: intravenous; LAC: lupus anticoagulant; NA: not applicable; s.d.: standard deviation; SDI: Systemic Lupus International Collaborating Clinics (SLICC)/American College of Rheumatology (ACR) Damage Index; SLE: systemic lupus erythematosus; SLEDAI-2K: Systemic Lupus Erythematosus Disease Activity Index 2000; UPCR: urinary protein/creatinine ratio.

*Alaska Native or American Indian from North, South or Central America.
[†]Hydroxychloroquine, chloroquine, mepacrine, mepacrine hydrochloride or quinine sulfate.

Supplementary Table S2. Comparisons between patients who developed at least one renal flare and patients who did not during follow-up in the subgroup of patients treated with IV belimumab 10 mg/kg.

	All patients (N=602)	Renal flare (N=48)	No renal flare (N=554)	*P* value
**Patient characteristics**				
Age; mean (s.d.)	37.6 (11.3)	36.4 (10.5)	37.7 (11.4)	0.478
Female sex; n (%)	584 (97.0)	46 (95.8)	538 (97.1)	0.954
Ancestries; n (%)				
Asian	180 (29.9)	22 (45.8)	158 (28.5)	**0.019**
Black/African American	184 (30.6)	11 (22.9)	173 (31.2)	0.300
Indigenous American*	68 (11.3)	3 (6.2)	65 (11.7)	0.361
White/Caucasian	170 (28.2)	12 (25.0)	158 (28.5)	0.724
**Clinical data at baseline**				
SLE duration (years); median (IQR)	3.9 (1.5–8.5); N=601	3.5 (1.1–8.5)	3.9 (1.5–8.5); N=553	0.676
Mean BMI (week 0–52); mean (s.d.)	26.1 (6.6); N=565	24.7 (5.8); N=42	26.2 (6.6); N=523	0.135
SLEDAI-2K; mean (s.d.)	9.4 (3.1)	8.3 (2.9)	9.5 (3.1)	**0.006**
Extra renal cSLEDAI-2K; mean (s.d.)	7.1 (3.0)	5.8 (2.5)	7.2 (3.0)	**0.001**
SDI score; median (IQR)	0.0 (0.0–1.0); N=601	0.0 (0.0–1.0)	0.0 (0.0–1.0); N=553	0.713
SDI score ≥1; n (%)	201 (33.4); N=601	17 (35.4)	184 (33.3); N=553	0.887
**Serological profile at baseline**				
Anti-dsDNA (+); n (%)	379 (63.0)	34 (70.8)	345 (62.3)	0.307
Anti-Sm (+); n (%)				
at baseline	82 (25.8); N=318	7 (29.2); N=24	75 (25.5); N=294	0.880
ever	82 (25.8); N=318	7 (29.2); N=24	75 (25.5); N=294	0.880
Anti-RNP (+); n (%)				
at baseline	54 (36.0); N=9	3 (33.3); N=9	51 (36.2); N=141	1.000
ever	54 (36.0); N=9	3 (33.3); N=9	51 (36.2); N=141	1.000
Anti-ribosomal P (+); n (%)				
at baseline	135 (29.3); N=460	11 (33.3); N=33	124 (29.0); N=427	0.746
ever	135 (29.3); N=460	11 (33.3); N=33	124 (29.0); N=427	0.746
aPL (+); n (%)				
aCL				
aCL IgA	9 (1.6); N=563	2 (4.3); N=46	7 (1.4); N=517	0.348
aCL IgG	67 (11.9); N=564	7 (15.2); N=46	60 (11.6); N=518	0.622
aCL IgM	64 (11.3); N=564	6 (13.0); N=46	58 (11.2); N=518	0.892
Anti-β_2-GPI				
anti-β_2-GPI IgA	23 (15.1); N=152	3 (33.3); N=9	20 (14.0); N=143	0.275
anti-β_2-GPI IgG	2 (1.3); N=152	0 (0.0); N=9	2 (1.4); N=143	1.000
anti-β_2-GPI IgM	12 (7.9); N=152	0 (0.0); N=9	12 (8.4); N=143	0.788

LAC	19 (12.9); N=147	0 (0.0); N=9	19 (13.8); N=138	0.496
aPL (+) ever	170 (30.1); N=564	14 (30.4); N=46	156 (30.1); N=518	1.000
BAFF (ng/mL); mean (s.d.)	1.45 (1.03); N=441	1.24 (0.79); N=38	1.47 (1.05); N=403	0.078
Low C3 levels; n (%)	252 (41.9)	23 (47.9)	229 (41.3)	0.463
Low C4 levels; n (%)	231 (38.4)	21 (43.8)	210 (37.9)	0.520
**Renal markers at baseline**				
Serum albumin (g/L); mean (s.d.)	41.39 (3.67)	41.83 (4.65)	41.35 (3.57)	0.499
Serum creatinine (μmol/L); mean (s.d.)	65.37 (14.21)	67.67 (17.99)	65.17 (13.84)	0.763
UPCR (mg/mg); mean (s.d.)	0.13 (0.08)	0.13 (0.10)	0.13 (0.08)	0.842
eGFR (mL/min); mean (s.d.)	115.41 (35.28); N=601	111.81 (39-51)	115.73 (34.91); N=553	0.371
**Medications**				
Prednisone equivalent dose during follow-up	9.90 (7.70)	11.72 (10.04)	9.75 (7.45)	0.363
Treatment at baseline; n (%)				
Antimalarial agents[†]	406 (67.4)	33 (68.8)	373 (67.3)	0.967
Immunosuppressants				
Azathioprine	116 (19.3)	7 (14.6)	109 (19.7)	0.505
Methotrexate	82 (13.6)	5 (10.4)	77 (13.9)	0.649
Mycophenolic acid	60 (10.0)	11 (22.9)	49 (8.8)	**0.004**
Oral cyclophosphamide	7 (1.2)	2 (4.2)	5 (0.9)	0.186
Tacrolimus	10 (1.7)	1 (2.1)	9 (1.6)	1.000
Cyclosporine	19 (3.2)	2 (4.2)	17 (3.1)	1.000
Leflunomide	18 (3.0)	1 (2.1)	17 (3.1)	1.000

Data are presented as numbers (percentage) or means (standard deviation). In case of non-normal distributions, the medians (interquartile range) are indicated. In case of missing values, numbers of patients with available data are indicated. Statistically significant *P* values are in bold.

(+): positive levels; aCL: anti cardiolipin antibodies; anti-β_2-GPI: anti-β2-glycoprotein I antibodies; anti-dsDNA: anti-double-stranded DNA antibodies; anti-RNP: anti-ribonucleoprotein antibodies; anti-Sm: anti-Smith antibodies; aPL: antiphospholipid antibodies; BAFF: B cell activating factor belonging to the TNF ligand family; BMI: body mass index; C3: complement component 3; C4: complement component 4; cSLEDAI-2K: clinical SLEDAI-2K; eGFR: estimated glomerular filtration rate; Ig: immunoglobulin; IQR: interquartile range; IV: intravenous; LAC: lupus anticoagulant; s.d.: standard deviation; SDI: Systemic Lupus International Collaborating Clinics (SLICC)/American College of Rheumatology (ACR) Damage Index; SLE: systemic lupus erythematosus; SLEDAI-2K: Systemic Lupus Erythematosus Disease Activity Index 2000; UPCR: urinary protein/creatinine ratio.

*Alaska Native or American Indian from North, South or Central America.
[†]Hydroxychloroquine, chloroquine, mepacrine, mepacrine hydrochloride or quinine sulfate.

Supplementary Table S3. Comparisons between patients who developed at least one renal flare and patients who did not during follow-up in the subgroup of patients treated with SC belimumab 200 mg.

	All patients (N=343)	Renal flare (N=18)	No renal flare (N=325)	*P* value
**Patient characteristics**				
Age; mean (s.d.)	39.8 (12.1)	46.1 (13.2)	39.5 (12.0)	0.066
Female sex; n (%)	327 (95.3)	17 (94.4)	310 (95.4)	1.000
Ancestries; n (%)				
Asian	44 (12.8)	1 (5.6)	43 (13.2)	0.558
Black/African American	29 (8.5)	4 (22.2)	25 (7.7)	0.085
Indigenous American*	29 (8.5)	1 (5.6)	28 (8.6)	0.985
White/Caucasian	241 (70.3)	12 (66.7)	229 (70.5)	0.938
**Clinical data at baseline**				
SLE duration (years); median (IQR)	4.3 (1.1–8.9)	3.0 (1.2–8.4)	4.3 (1.2–8.9)	0.876
Mean BMI (week 0–52); mean (s.d.)	26.6 (6.4); N=342	27.0 (5.7)	26.6 (6.5); N=324	0.482
SLEDAI-2K; mean (s.d.)	10.2 (2.6)	9.4 (1.7)	10.2 (2.6)	0.301
Extra renal cSLEDAI-2K; mean (s.d.)	8.1 (2.6)	7.4 (1.4)	8.1 (2.7)	0.281
SDI score; median (IQR)	0.0 (0.0–1.0)	1.0 (0.0–1.8)	0.0 (0.0–1.0)	**0.012**
SDI score ≥1; n (%)	121 (35.3)	11 (61.1)	110 (33.8)	**0.035**
**Serological profile at baseline**				
Anti-dsDNA (+); n (%)	215 (62.7)	12 (66.7)	203 (62.5)	0.913
Anti-Sm (+); n (%)				
at baseline	77 (22.8); N=337	7 (38.9)	70 (21.9); N=319	0.168
ever	78 (23.1); N=338	7 (38.9)	71 (22.2); N=320	0.177
Anti-RNP (+); n (%)				
at baseline	94 (28.1); N=335	4 (23.5); N=17	90 (28.3); N=318	0.881
ever	95 (28.1); N=338	4 (22.2)	91 (28.4); N=320	0.763
Anti-ribosomal P (+); n (%)				
at baseline	71 (21.1); N=337	1 (5.6)	70 (21.9); N=319	0.173
ever	71 (21.1); N=337	1 (5.6)	70 (21.9); N=319	0.173
aPL (+); n (%)				
aCL				
aCL IgA	10 (2.9); N=340	2 (11.1)	8 (2.5); N=322	0.164
aCL IgG	26 (7.6); N=340	2 (11.1)	24 (7.5); N=322	0.910
aCL IgM	50 (14.7); N=340	2 (11.1)	48 (14.9); N=322	0.920
Anti-β_2-GPI				
anti-β_2-GPI IgA	55 (16.4); N=336	3 (16.7)	52 (16.4); N=318	1.000
anti-β_2-GPI IgG	10 (3.0); N=336	1 (5.6)	9 (2.8); N=318	1.000
anti-β_2-GPI IgM	26 (7.7); N=336	2 (11.1)	24 (7.5); N=318	0.923

LAC	73 (21.9); N=334	4 (22.2)	69 (21.8); N=316	1.000
aPL (+) ever	207 (60.3)	9 (5.0)	198 (60.9)	0.500
BAFF (ng/mL); mean (s.d.)	1.46 (1.04); N=341	1.45 (0.60)	1.46 (1.06); N=323	0.585
Low C3 levels; n (%)	126 (36.7)	6 (33.3)	120 (36.9)	0.955
Low C4 levels; n (%)	85 (24.8)	4 (22.2)	81 (24.9)	1.000
**Renal markers at baseline**				
Serum albumin (g/L); mean (s.d.)	42.10 (3.68)	40.89 (5.28)	42.16 (1.06)	0.485
Serum creatinine (μmol/L); mean (s.d.)	65.18 (13.83)	75.17 (18.93)	64.63 (13.32)	**0.007**
UPCR (mg/mg); mean (s.d.)	0.12 (0.07)	0.14 (0.11)	0.11 (0.07)	0.515
eGFR (mL/min); mean (s.d.)	117.36 (38.31)	103.78 (42.74)	118.11 (37.98)	0.059
**Medications**				
Prednisone equivalent dose during follow-up	9.52 (7.32)	10.42 (9.35)	9.47 (7.21)	0.852
Treatment at baseline; n (%)				
Antimalarial agents†	236 (68.8)	6 (33.3)	230 (70.8)	**0.002**
Immunosuppressants				
Azathioprine	65 (19.0)	2 (11.1)	63 (19.4)	0.573
Methotrexate	38 (11.1)	1 (5.6)	37 (11.4)	0.703
Mycophenolic acid	24 (7.0)	3 (16.7)	21 (6.5)	0.239
Oral cyclophosphamide	3 (0.9)	2 (11.1)	1 (0.3)	**< 0.001**
Tacrolimus	9 (2.6)	1 (5.6)	8 (2.5)	0.967
Cyclosporine	8 (2.3)	0 (0.0)	8 (2.5)	1.000
Leflunomide	4 (1.2)	1 (5.6)	3 (0.9)	0.513

Data are presented as numbers (percentage) or means (standard deviation). In case of non-normal distributions, the medians (interquartile range) are indicated. In case of missing values, numbers of patients with available data are indicated. Statistically significant *P* values are in bold.

(+): positive levels; aCL: anti cardiolipin antibodies; anti-β_2-GPI: anti-β2-glycoprotein I antibodies; anti-dsDNA: anti-double-stranded DNA antibodies; anti-RNP: anti-ribonucleoprotein antibodies; anti-Sm: anti-Smith antibodies; aPL: antiphospholipid antibodies; BAFF: B cell activating factor belonging to the TNF ligand family; BMI: body mass index; C3: complement component 3; C4: complement component 4; cSLEDAI-2K: clinical SLEDAI-2K; eGFR: estimated glomerular filtration rate; Ig: immunoglobulin; IQR: interquartile range; LAC: lupus anticoagulant; SC: subcutaneous; s.d.: standard deviation; SDI: Systemic Lupus International Collaborating Clinics (SLICC)/American College of Rheumatology (ACR) Damage Index; SLE: systemic lupus erythematosus; SLEDAI-2K: Systemic Lupus Erythematosus Disease Activity Index 2000; UPCR: urinary protein/creatinine ratio.

*Alaska Native or American Indian from North, South or Central America.
†Hydroxychloroquine, chloroquine, mepacrine, mepacrine hydrochloride or quinine sulfate.

Supplementary Table S4. Comparisons between patients who developed at least one renal flare and patients who did not during follow-up in the subgroup of placebo recipients.

	All patients (N=588)	Renal flare (N=57)	No renal flare (N=531)	*P* value
**Patient characteristics**				
Age; mean (s.d.)	38.6 (12.2)	33.8 (10.6)	39.1 (12.2)	**0.002**
Female sex; n (%)	564 (95.9)	56 (98.2)	508 (95.7)	0.560
Ancestries; n (%)				
Asian	131 (22.3)	17 (29.8)	114 (21.5)	0.203
Black/African American	99 (16.8)	9 (15.8)	90 (16.9)	0.971
Indigenous American*	81 (13.8)	10 (17.5)	71 (13.4)	0.505
White/Caucasian	277 (47.1)	21 (36.8)	256 (48.2)	0.135
**Clinical data at baseline**				
SLE duration (years); median (IQR)	4.2 (1.5–9.6)	3.3 (1.5–9.2)	4.4 (1.5–9.6)	0.567
Mean BMI (week 0–52); mean (s.d.)	26.0 (6.2); N=575	24.8 (5.3); N=53	26.2 (6.3); N=522	0.143
SLEDAI-2K; mean (s.d.)	9.4 (2.9)	9.9 (3.8)	9.3 (2.7)	0.768
Extra renal cSLEDAI-2K; mean (s.d.)	7.1 (2.8)	6.8 (3.8)	7.2 (2.6)	0.054
SDI score; median (IQR)	0.0 (0.0–1.0)	0.0 (0.0–1.0)	0.0 (0.0–1.0)	0.050
SDI score ≥1; n (%)	223 (37.9)	15 (26.3)	208 (39.2)	0.079
**Serological profile at baseline**				
Anti-dsDNA (+); n (%)	368 (62.6)	47 (82.5)	321 (60.5)	**0.002**
Anti-Sm (+); n (%)				
at baseline	113 (24.2); N=467	14 (36.8); N=38	99 (23.1); N=429	0.089
ever	115 (24.6); N=468	14 (36.8); N=38	101 (23.5); N=430	0.102
Anti-RNP (+); n (%)				
at baseline	58 (25.9); N=224	13 (61.9); N=21	45 (22.2); N=203	**< 0.001**
ever	58 (25.9); N=224	13 (61.9); N=21	45 (22.2); N=203	**< 0.001**
Anti-ribosomal P (+); n (%)				
at baseline	149 (28.9); N=515	23 (48.9); N=47	126 (26.9); N=468	**0.003**
ever	149 (28.9); N=515	23 (48.9); N=47	126 (26.9); N=468	**0.003**
aPL (+); n (%)				
aCL				
aCL IgA	12 (2.1); N=569	2 (3.5)	10 (2.0); N=512	0.772
aCL IgG	81 (14.2); N=569	12 (21.1)	69 (13.5); N=512	0.176
aCL IgM	44 (7.7); N=569	4 (7.0)	40 (7.8); N=512	1.000
Anti-β_2-GPI				
anti-β_2-GPI IgA	39 (17.3); N=226	6 (28.6); N=21	33 (16.1); N=205	0.255
anti-β_2-GPI IgG	11 (4.9); N=226	1 (4.8); N=21	10 (4.9); N=205	1.000
anti-β_2-GPI IgM	11 (4.9); N=226	1 (4.8); N=21	10 (4.9); N=205	1.000
LAC	42 (18.7); N=225	1 (4.8); N=21	41 (20.1); N=204	0.155

aPL (+) ever	216 (37.9); N=570	26 (45.6)	190 (37.0); N=513	0.262
BAFF (ng/mL); mean (s.d.)	1.43 (0.92); N=526	1.46 (0.96); N=48	1.42 (0.92); N=478	0.954
Low C3 levels; n (%)	229 (38.9)	36 (63.2)	193 (36.3)	**< 0.001**
Low C4 levels; n (%)	210 (35.7)	32 (56.1)	178 (33.5)	0.001
**Renal markers at baseline**				
Serum albumin (g/L); mean (s.d.)	41.44 (3.66)	40.16 (2.94)	41.58 (3.70)	**0.003**
Serum creatinine (μmol/L); mean (s.d.)	66.65 (14.55)	65.44 (18.97)	66.79 (14.01)	**0.041**
UPCR (mg/mg); mean (s.d.)	0.12 (0.07)	0.13 (0.06)	0.12 (0.07)	**0.012**
eGFR (mL/min); mean (s.d.)	112.04 (34.25)	115.40 (36.89)	111.68 (33.98)	0.266
**Medications**				
Prednisone equivalent dose during follow-up	10.42 (8.39)	14.60 (8.49)	9.98 (8.27)	**< 0.001**
Treatment at baseline; n (%)				
Antimalarial agents[†]	416 (70.7)	43 (75.4)	373 (70.2)	0.505
Immunosuppressants				
Azathioprine	107 (18.2)	12 (21.1)	95 (17.9)	0.684
Methotrexate	105 (17.9)	4 (7.0)	101 (19.0)	**0.039**
Mycophenolic acid	46 (7.8)	10 (17.5)	36 (6.8)	**0.009**
Oral cyclophosphamide	6 (1.0)	2 (3.5)	4 (0.8)	0.203
Tacrolimus	17 (2.9)	0 (0.0)	17 (3.2)	0.340
Cyclosporine	17 (2.9)	1 (1.8)	16 (3.0)	0.902
Leflunomide	14 (2.4)	3 (5.3)	11 (2.1)	0.296

Data are presented as numbers (percentage) or means (standard deviation). In case of non-normal distributions, the medians (interquartile range) are indicated. In case of missing values, numbers of patients with available data are indicated. Statistically significant *P* values are in bold.

(+): positive levels; aCL: anti cardiolipin antibodies; anti-β_2-GPI: anti-β2-glycoprotein I antibodies; anti-dsDNA: anti-double-stranded DNA antibodies; anti-RNP: anti-ribonucleoprotein antibodies; anti-Sm: anti-Smith antibodies; aPL: antiphospholipid antibodies; BAFF: B cell activating factor belonging to the TNF ligand family; BMI: body mass index; C3: complement component 3; C4: complement component 4; cSLEDAI-2K: clinical SLEDAI-2K; eGFR: estimated glomerular filtration rate; Ig: immunoglobulin; IQR: interquartile range; LAC: lupus anticoagulant; s.d.: standard deviation; SDI: Systemic Lupus International Collaborating Clinics (SLICC)/American College of Rheumatology (ACR) Damage Index; SLE: systemic lupus erythematosus; SLEDAI-2K: Systemic Lupus Erythematosus Disease Activity Index 2000; UPCR: urinary protein/creatinine ratio.

*Alaska Native or American Indian from North, South or Central America.
[†]Hydroxychloroquine, chloroquine, mepacrine, mepacrine hydrochloride or quinine sulfate.

Supplementary Table S5. Demographics and comparisons in the subgroup of belimumab-treated patients, stratified by belimumab dosage forms.

	IV BLM 1 mg/kg (N=331)	IV BLM 10 mg/kg (N=602)	SC BLM (N=343)	*P* value
**Patient characteristics**				
Age; mean (s.d.)	39.2 (11.6)	37.6 (11.3)	39.5 (12.0)	**0.014**
Female sex; n (%)	294 (94.5)	584 (97.0)	310 (95.4)	0.160
Ancestries; n (%)				
Asian	41 (13.2)	180 (29.9)	43 (13.2)	**< 0.001**
Black/African American	27 (8.7)	184 (30.6)	25 (7.7)	**< 0.001**
Indigenous American*	65 (20.9)	68 (11.3)	28 (8.6)	**< 0.001**
White/Caucasian	178 (57.2)	170 (28.2)	229 (70.5)	**< 0.001**
**Clinical data at baseline**				
SLE duration (years); median (IQR)	4.5 (1.1–9.2)	3.9 (1.5–8.5); N=601	4.3 (1.1–8.9)	0.980
Mean BMI (week 0–52); mean (s.d.)	26.6 (6.4)	26.1 (6.6); N=565	26.6 (6.4); N=342	0.174
SLEDAI-2K; mean (s.d.)	9.2 (3.3)	9.4 (3.1)	10.2 (2.6)	**< 0.001**
Extra renal cSLEDAI-2K; mean (s.d.)	6.8 (3.0)	7.1 (3.0)	8.1 (2.6)	**< 0.001**
SDI score; median (IQR)	0.0 (0.0–1.0); N=310	0.0 (0.0–1.0); N=601	0.0 (0.0–1.0)	**0.021**
SDI score ≥1; n (%)	130 (41.9); N=310	201 (33.4); N=601	121 (35.3)	**0.038**
**Serological profile at baseline**				
Anti-dsDNA (+); n (%)	191 (61.4)	379 (63.0)	215 (62.7)	0.898
Anti-Sm (+); n (%)				
at baseline	76 (24.4)	82 (25.8); N=318	77 (22.8); N=337	0.680
ever	76 (24.4)	82 (25.8); N=318	78 (23.1); N=338	0.722
Anti-RNP (+); n (%)				
at baseline	NA	54 (36.0); N=150	94 (28.1); N=335	NA
ever	NA	54 (36.0); N=150	95 (28.1); N=338	NA
Anti-ribosomal P (+); n (%)				
at baseline	108 (35.4); N=305	135 (29.3); N=460	71 (21.1); N=337	**< 0.001**
ever	108 (35.4); N=305	135 (29.3); N=460	71 (21.1); N=337	**< 0.001**
aPL (+); n (%)				
aCL				
aCL IgA	3 (1.0); N=309	9 (1.6); N=563	10 (2.9); N=340	0.149
aCL IgG	69 (22.2)	67 (11.9); N=564	26 (7.6); N=340	**< 0.001**
aCL IgM	24 (7.7)	64 (11.3); N=564	50 (14.7); N=340	**0.019**
Anti-β_2-GPI				
anti-β_2-GPI IgA	NA	23 (15.1); N=152	55 (16.4); N=336	NA
anti-β_2-GPI IgG	NA	2 (1.3); N=152	10 (3.0); N=336	NA
anti-β_2-GPI IgM	NA	12 (7.9); N=152	26 (7.7); N=336	NA
LAC	NA	19 (12.9); N=147	73 (21.9); N=334	NA

aPL (+) ever	81 (26.0)	170 (30.1); N=564	207 (60.3)	**< 0.001**
BAFF (ng/mL); mean (s.d.)	1.64 (1.20); N=309	1.45 (1.03); N=441	1.46 (1.04); N=341	**0.005**
Low C3 levels; n (%)	125 (40.2)	252 (41.9)	126 (36.7)	0.302
Low C4 levels; n (%)	160 (51.4)	231 (38.4)	85 (24.8)	**< 0.001**
**Renal markers at baseline**				
Serum albumin (g/L); mean (s.d.)	41.21 (3.83)		42.10 (3.68)	**0.002**
Serum creatinine (μmol/L); mean (s.d.)	70.17 (13.37)		65.18 (13.83)	**< 0.001**
UPCR (mg/mg); mean (s.d.)	0.12 (0.07)		0.12 (0.07)	**0.028**
eGFR (mL/min); mean (s.d.)	107.25 (32.64)		117.36 (38.31)	**< 0.001**
**Medications**				
Prednisone equivalent dose during follow-up	9.59 (7.70)	9.90 (7.70)	9.52 (7.32)	0.595
Treatment at baseline; n (%)				
Antimalarial agents†	200 (64.3)	406 (67.4)	236 (68.8)	0.454
Immunosuppressants				
Azathioprine	64 (20.6)	116 (19.3)	65 (19.0)	0.853
Methotrexate	53 (17.0)	82 (13.6)	38 (11.1)	0.086
Mycophenolic acid	21 (6.8)	60 (10.0)	24 (7.0)	0.142
Oral cyclophosphamide	5 (1.6)	7 (1.2)	3 (0.9)	0.686
Tacrolimus	4 (1.3)	10 (1.7)	9 (2.6)	0.405
Cyclosporine	9 (2.9)	19 (3.2)	8 (2.3)	0.766
Leflunomide	7 (2.3)	18 (3.0)	4 (2.1)	0.199

Data are presented as numbers (percentage) or means (standard deviation). In case of non-normal distributions, the medians (interquartile range) are indicated. In case of missing values, numbers of patients with available data are indicated. Statistically significant *P* values are in bold.

(+): positive levels; aCL: anti cardiolipin antibodies; anti-β_2-GPI: anti-β2-glycoprotein I antibodies; anti-dsDNA: anti-double-stranded DNA antibodies; anti-RNP: anti-ribonucleoprotein antibodies; anti-Sm: anti-Smith antibodies; aPL: antiphospholipid antibodies; BAFF: B cell activating factor belonging to the TNF ligand family; BLM: belimumab; BMI: body mass index; C3: complement component 3; C4: complement component 4; cSLEDAI-2K: clinical SLEDAI-2K; eGFR: estimated glomerular filtration rate; Ig: immunoglobulin; IQR: interquartile range; IV: intravenous; LAC: lupus anticoagulant; NA: not applicable; SC: subcutaneous; s.d.: standard deviation; SDI: Systemic Lupus International Collaborating Clinics (SLICC)/American College of Rheumatology (ACR) Damage Index; SLE: systemic lupus erythematosus; SLEDAI-2K: Systemic Lupus Erythematosus Disease Activity Index 2000; UPCR: urinary protein/creatinine ratio.

*Alaska Native or American Indian from North, South or Central America.
†Hydroxychloroquine, chloroquine, mepacrine, mepacrine hydrochloride or quinine sulfate.

PRISMA-IPD Checklist of items to include when reporting a systematic review and meta-analysis of individual participant data (IPD)

PRISMA-IPD Section/topic	Item No	Checklist item	Reported on page
**Title**			
Title	1	Identify the report as a systematic review and meta-analysis of individual participant data.	NA
**Abstract**			
Structured summary	2	Provide a structured summary including as applicable:	2
		**Background**: state research question and main objectives, with information on participants, interventions, comparators and outcomes.	
		**Methods**: report eligibility criteria; data sources including dates of last bibliographic search or elicitation, noting that IPD were sought; methods of assessing risk of bias.	
		**Results**: provide number and type of studies and participants identified and number (%) obtained; summary effect estimates for main outcomes (benefits and harms) with confidence intervals and measures of statistical heterogeneity. Describe the direction and size of summary effects in terms meaningful to those who would put findings into practice.	
		**Discussion:** state main strengths and limitations of the evidence, general interpretation of the results and any important implications.	
		**Other:** report primary funding source, registration number and registry name for the systematic review and IPD meta-analysis.	
**Introduction**			
Rationale	3	Describe the rationale for the review in the context of what is already known.	3
Objectives	4	Provide an explicit statement of the questions being addressed with reference, as applicable, to participants, interventions, comparisons, outcomes and study design (PICOS). Include any hypotheses that relate to particular types of participant-level subgroups.	3
**Methods**			
Protocol and registration	5	Indicate if a protocol exists and where it can be accessed. If available, provide registration information including registration number and registry name. Provide publication details, if applicable.	3

Eligibility criteria	6	Specify inclusion and exclusion criteria including those relating to participants, interventions, comparisons, outcomes, study design and characteristics (e.g. years when conducted, required minimum follow-up). Note whether these were applied at the study or individual level i.e. whether eligible participants were included (and ineligible participants excluded) from a study that included a wider population than specified by the review inclusion criteria. The rationale for criteria should be stated.	3–4
Identifying studies - information sources	7	Describe all methods of identifying published and unpublished studies including, as applicable: which bibliographic databases were searched with dates of coverage; details of any hand searching including of conference proceedings; use of study registers and agency or company databases; contact with the original research team and experts in the field; open adverts and surveys. Give the date of last search or elicitation.	NA
Identifying studies - search	8	Present the full electronic search strategy for at least one database, including any limits used, such that it could be repeated.	NA
Study selection processes	9	State the process for determining which studies were eligible for inclusion.	NA
Data collection processes	10	Describe how IPD were requested, collected and managed, including any processes for querying and confirming data with investigators. If IPD were not sought from any eligible study, the reason for this should be stated (for each such study).	4
		If applicable, describe how any studies for which IPD were not available were dealt with. This should include whether, how and what aggregate data were sought or extracted from study reports and publications (such as extracting data independently in duplicate) and any processes for obtaining and confirming these data with investigators.	
Data items	11	Describe how the information and variables to be collected were chosen. List and define all study level and participant level data that were sought, including baseline and follow-up information. If applicable, describe methods of standardising or translating variables within the IPD datasets to ensure common scales or measurements across studies.	5–6
IPD integrity	A1	Describe what aspects of IPD were subject to data checking (such as sequence generation, data consistency and completeness, baseline imbalance) and how this was done.	4
Risk of bias assessment in individual studies.	12	Describe methods used to assess risk of bias in the individual studies and whether this was applied separately for each outcome. If applicable, describe how findings of IPD checking were used to inform the assessment. Report if and how risk of bias assessment was used in any data synthesis.	NA
Specification of outcomes and	13	State all treatment comparisons of interests. State all outcomes addressed and define them in detail. State whether they were pre-specified for the review and, if applicable, whether they were primary/main or secondary/additional outcomes. Give the	5–7

effect measures		principal measures of effect (such as risk ratio, hazard ratio, difference in means) used for each outcome.	
Synthesis methods	14	Describe the meta-analysis methods used to synthesise IPD. Specify any statistical methods and models used. Issues should include (but are not restricted to):<br>• Use of a one-stage or two-stage approach.<br>• How effect estimates were generated separately within each study and combined across studies (where applicable).<br>• Specification of one-stage models (where applicable) including how clustering of patients within studies was accounted for.<br>• Use of fixed or random effects models and any other model assumptions, such as proportional hazards.<br>• How (summary) survival curves were generated (where applicable).<br>• Methods for quantifying statistical heterogeneity (such as I^2 and τ^2).<br>• How studies providing IPD and not providing IPD were analysed together (where applicable).<br>• How missing data within the IPD were dealt with (where applicable).	7
Exploration of variation in effects	A2	If applicable, describe any methods used to explore variation in effects by study or participant level characteristics (such as estimation of interactions between effect and covariates). State all participant-level characteristics that were analysed as potential effect modifiers, and whether these were pre-specified.	7
Risk of bias across studies	15	Specify any assessment of risk of bias relating to the accumulated body of evidence, including any pertaining to not obtaining IPD for particular studies, outcomes or other variables.	NA
Additional analyses	16	Describe methods of any additional analyses, including sensitivity analyses. State which of these were pre-specified.	NA
**Results**			
Study selection and IPD obtained	17	Give numbers of studies screened, assessed for eligibility, and included in the systematic review with reasons for exclusions at each stage. Indicate the number of studies and participants for which IPD were sought and for which IPD were obtained. For those studies where IPD were not available, give the numbers of studies and participants for which aggregate data were available. Report reasons for non-availability of IPD. Include a flow diagram.	PRISMA Flow Diagram
Study characteristics	18	For each study, present information on key study and participant characteristics (such as description of interventions, numbers of participants, demographic data, unavailability of outcomes, funding source, and if applicable duration of follow-up). Provide (main) citations for each study. Where applicable, also report similar study characteristics for any studies not providing IPD.	Table 1

IPD integrity	A3	Report any important issues identified in checking IPD or state that there were none.	Table 1
Risk of bias within studies	19	Present data on risk of bias assessments. If applicable, describe whether data checking led to the up-weighting or down-weighting of these assessments. Consider how any potential bias impacts on the robustness of meta-analysis conclusions.	NA
Results of individual studies	20	For each comparison and for each main outcome (benefit or harm), for each individual study report the number of eligible participants for which data were obtained and show simple summary data for each intervention group (including, where applicable, the number of events), effect estimates and confidence intervals. These may be tabulated or included on a forest plot.	NA
Results of syntheses	21	Present summary effects for each meta-analysis undertaken, including confidence intervals and measures of statistical heterogeneity. State whether the analysis was pre-specified, and report the numbers of studies and participants and, where applicable, the number of events on which it is based.	7–9
		When exploring variation in effects due to patient or study characteristics, present summary interaction estimates for each characteristic examined, including confidence intervals and measures of statistical heterogeneity. State whether the analysis was pre-specified. State whether any interaction is consistent across trials.	
		Provide a description of the direction and size of effect in terms meaningful to those who would put findings into practice.	
Risk of bias across studies	22	Present results of any assessment of risk of bias relating to the accumulated body of evidence, including any pertaining to the availability and representativeness of available studies, outcomes or other variables.	NA
Additional analyses	23	Give results of any additional analyses (e.g. sensitivity analyses). If applicable, this should also include any analyses that incorporate aggregate data for studies that do not have IPD. If applicable, summarise the main meta-analysis results following the inclusion or exclusion of studies for which IPD were not available.	NA
**Discussion**			
Summary of evidence	24	Summarise the main findings, including the strength of evidence for each main outcome.	9
Strengths and limitations	25	Discuss any important strengths and limitations of the evidence including the benefits of access to IPD and any limitations arising from IPD that were not available.	11

Conclusions	26	Provide a general interpretation of the findings in the context of other evidence.	9–11
Implications	A4	Consider relevance to key groups (such as policy makers, service providers and service users). Consider implications for future research.	9–11
**Funding**			
Funding	27	Describe sources of funding and other support (such as supply of IPD), and the role in the systematic review of those providing such support.	11–12

A1 – A3 denote new items that are additional to standard PRISMA items. A4 has been created as a result of re-arranging content of the standard PRISMA statement to suit the way that systematic review IPD meta-analyses are reported.

© Reproduced with permission of the PRISMA IPD Group, which encourages sharing and reuse for non-commercial purpose

PRISMA IPD Flow Diagram

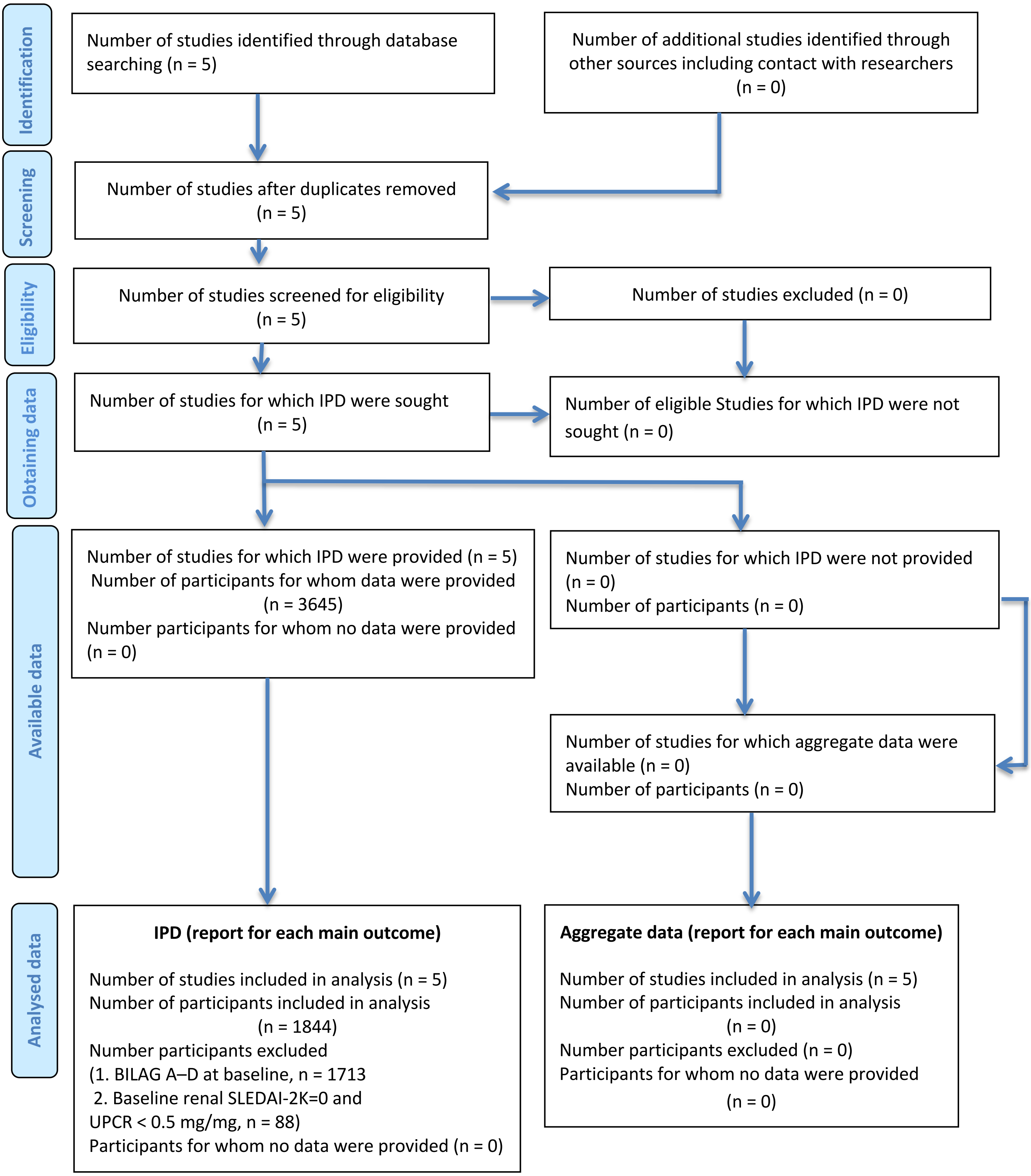

The PRISMA IPD flow diagram

© Reproduced with permission of the PRISMA IPD Group, which encourages sharing and reuse for non commercial purposes
